# MiR-495 inhibits esophageal squamous cell carcinoma progression by targeting Akt1

**DOI:** 10.18632/oncotarget.9981

**Published:** 2016-06-13

**Authors:** Yu Mao, Liang Li, Jia Liu, Le Wang, Yan Zhou

**Affiliations:** ^1^ Department of Oncology, The First Hospital of Qinhuangdao, Qinhuangdao, Hebei, China; ^2^ Department of Ultrasound, Beijing Anzhen Hospital, Capital Medical University, Beijing, China; ^3^ Institute of Basic Medical Sciences, Qilu Hospital, Shandong University, Jinan, China; ^4^ Department of Neurosurgery, Tianjin Medical University General Hospital, Tianjin Neurological Institute and Laboratory of Neuro -Oncology, Tianjin, China; ^5^ Department of Radiotherapy, Tianjin General Hospital, Tianjin, China

**Keywords:** miR-495, esophageal squamous cell carcinoma, progression, Akt1

## Abstract

MicroRNAs are involved in tumor initiation and progression by regulating oncogenes and tumor suppressor genes. Here we found that miR-495 are lower in clinical ESCC tissues than in adjacent non-tumor tissues. Moreover, the lower miR-495 expression correlated with increased lymph node metastasis (LNM), invasion and TNM stage. miR-495 overexpression predicted a favorable outcome in ESCC patients. miR-495 targeted a site in the 3′-UTR of Akt1, and miR-495 levels correlated inversely with Akt1 protein levels in ESCC tissue samples. Overexpression of miR-495 suppressed cell proliferation, blocked G1/S phase transition, and decreased migration and invasion by two ESCC cell lines *in vitro* and *in vivo*. Restoration of Akt1 protein levels in miR-495-overexpressing ESCC cells attenuated the inhibitory effects of miR-495. In addition, miR-495 suppressed cell cycle transition and the EMT signaling pathway through targeting Akt1, thereby inhibiting ESCC cell proliferation, migration, and invasion. Our results suggest that miR-495 may act as a tumor suppressor by targeting Akt1 in ESCC.

## INTRODUCTION

In East Asia, esophageal squamous cell carcinoma (ESCC) is the most predominant type of esophageal cancer [1]. Often diagnosed at later stages, the prognosis of patients with ESCC remains poor, with a 5-year survival rate of only 20–30% [2], despite recent advances in diagnostics and multimodal therapies. Tumor proliferation and metastasis are two main factors responsible for ESCC progression [3]. However, the molecular mechanisms of tumor progression and metastasis remain unclear [1]. Therefore, understanding the factors related to ESCC progression is necessary in the search for new prognostic biomarkers and therapeutic targets.

According to recent studies, microRNAs (miRNAs) which are small, endogenous, non-coding RNAs, 20–24 nucleotides in length, play crucial roles in numerous cancer-relevant biological processes, such as differentiation, proliferation, migration and apoptosis [4, 5]. MiRNAs generally function as the negative regulators of gene expression through binding to the complementary segments present in the 3′-untranslated region (3′-UTR) of target mRNAs and promoting the relative mRNA degradation or translation repression at the post-transcriptional level [6]. Deregulation of miRNAs has been reported to both activate and inhibit tumor progression [7]. For example, previous studies have demonstrated the dysregulation of miR-495 in multiple types of cancers, including pancreatic cancer [8], ovarian cancer [9], renal cancer [10], gastric cancer [11], hepatocellular cancer [12], and gallbladder cancer [13]. Furthermore, overexpression of miR-495 inhibits tumor progression by inducing G0/G1 cell cycle arrest [9] and by suppressing angiogenesis and metastasis [12]. However, the specific function of miR-495 in ESCC progression still remains unclear. Additionally, bioinformatic analysis predicts that miR-495 might regulate the expression of Akt1 protein by directly targeting the 3′-UTR of its mRNA, which plays a central role in the PI3K/AKT pathway [14]. Here, we demonstrated the down-regulation of miR-495 in ESCC specimens and its expression level was associated with increased lymph node metastasis (LNM), increased invasion, later TNM stages, and poor overall survival. Furthermore, we experimentally confirmed that miR-495 decreased tumor progression *in vitro* and *in vivo* by directly targeting Akt1. Besides, we have combined experimental and clinical studies to establish that upregulating miR-495 suppresses ESCC progression by inhibiting Akt1.

## RESULTS

### miR-495 is expressed at low levels in ESCC tissues and its association with clinical parameters in ESCC patients

**Figure 1:**
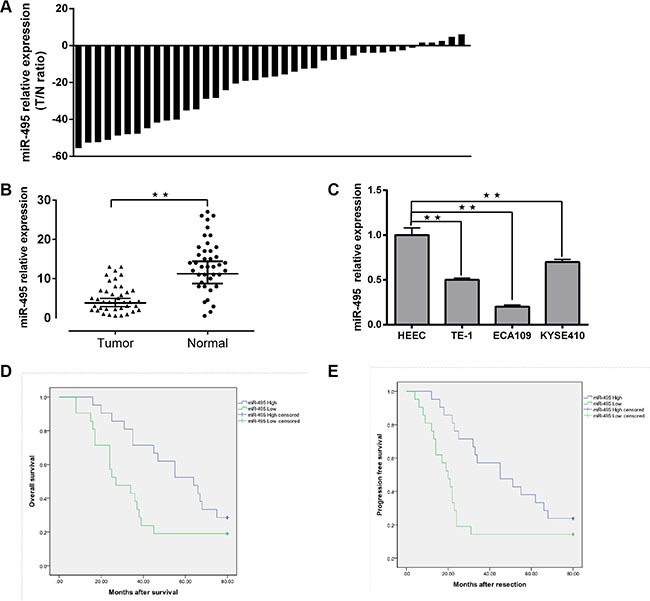
Downregulation of miR-495 in ESCC and its correlation with clinical parameters in ESCC patients (**A**) Expression of miR-495 in 40 pairs of ESCC tissues and the corresponding adjecent normal tissues. *U6* small nuclear RNA was used as an internal control. Data were analyzed using a ΔΔCt approach and expressed as log 2 fold change (ΔΔCt [ESCC/adjecent normal tissues]). (**B**) Expression of miR-495 in ESCC tissues and the corresponding adjecent normal tissues was compared using the Mann-Whitney *U*-test (**C**) Expression of miR-495 in three ESCC cell lines (ECA10, KYSE410 and TE-1) and normal esophageal epithelial cells. (**D, E**) Kaplan-Meier curves of OS and PFS for ESCC patients with high/low miR-495 expression. All values are the mean ± SD of triplicate measurements, and experiments were repeated 3 times with similar results. *P < 0.05, **P < 0.01.

Clinical tissues from 22 males and 18 females were used for qRT-PCR and their details are shown in Table 1. The 2-ΔΔCt method revealed that miR-495 expression was downregulated in 85% (34/40) of the ESCC tissues compared to the paired non-tumor tissues patients using a 2-fold change criterion (cutoff = −2) (Figure 1A). In general, miR-495 was significantly downregulated in ESCC tumor specimens in comparision with the adjacent non-tumor tissues (*p* < 0.01) (Figure 1B). Further study showed that significant associations were observed between lower miR-495vexpression levels and increased LNM, invasion, later TNM stages, and poor overall survival. (Table 2) (*p* < 0.05).

**Table 1:** Clinical characteristics of esophageal squamous cell carcinoma

Clinical parameter	*n*
Gender	
Male	22
Female	18
Age	
< 55	20
≥ 55	20
LNM	
N0	15
N1	19
N2	6
Invasion	
T1	8
T2	15
T3	12
T4a	5
Histological type	
Ulcerative	15
Medullary	9
Fungating	9
Others	7
TNM stage	
I	3
II a	9
II b	16
III	12

**Table 2:** Associations between expression levels of miR-495 and clinical parameters

	median ± SD	*P* value
Gender		0.754
Male	−19.4 ± **9.5**	
Female	−24.2 ± **8.4**	
Age		0.453
< 55	−19.5 ± **6.5**	
≥ 55	−23.6 ± **11.3**	
LNM		0.017
N0	−11.1 ± **5.1**	
N1	−21.1 ± **7.2**	
N2	−49.3 ± **6**	
Invasion		0.004
T1	−5.5 ± **3.7**	
T2	−15.9 ± **2.8**	
T3	−29.4 ± **6.4**	
T4a	−47.3 ± **8.9**	
Histological type		0.132
Ulcerative	−16.8 ± **8.8**	
Medullary	−15.2 ± **5.9**	
Fungating	−25.3 ± **6.7**	
Others	−35.0 ± **11.9**	
TNM stage		0.036
I	−0.3 ± **0.2**	
II a	−2.1 ± **1.1**	
II b	−19.5 ± **10.0**	
III	−44.3 ± **11.4**	

The miR-495 expression level of FFEP tumor tissues was measured using qRT-PCR, and relationships between miR-495 and clinical prognosis were explored. Our results showed that lower miR-495 down-regulation was correlated with shorter progression-free survival (PFS) (*P* = 0.01) and overall survival (OS) (*P* = 0.03) times. (Figure 1D, 1E).

We further compared the miR-495 expression levels of relative cell lines. The expression level of miR-495 was higher in human esophageal epithelial cell lines (HEEC) than in the ECA109, TE-1, and KYSE410 cell lines (Figure 1C).

### Ectopic miR-495 expression suppresses ESCC progression in both *vitro* and *vivo*

Firstly, the efficiency of miR-495 mimics or inhibitors transfection were measured. The results showed that intracellular miR-495 levels increased dramatically in cell lines transfected with mimics and decreased dramatically in cell lines transfected with inhibitors (Figure S1).

**Figure 2:**
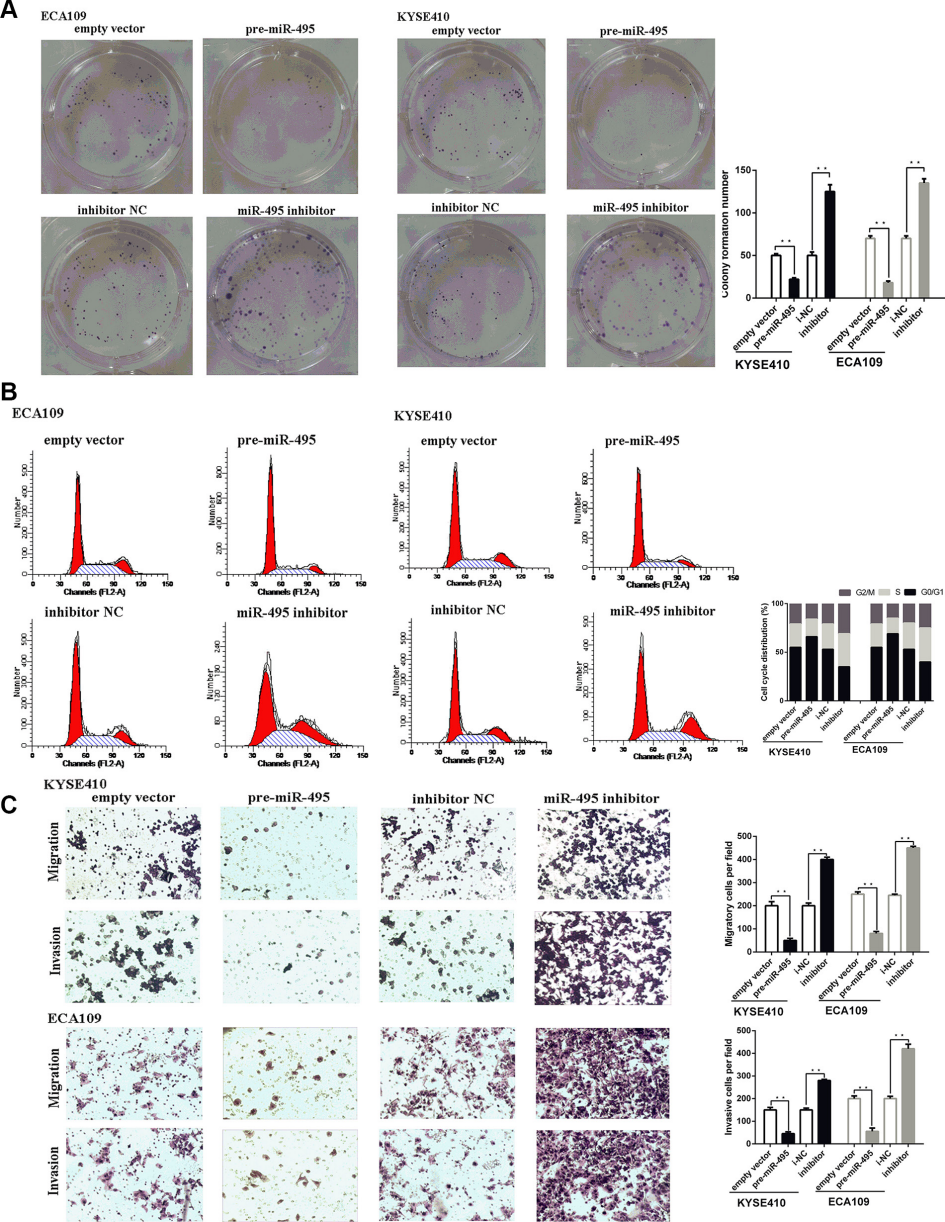
miR-495 inhibits ESCC progression *in vitro* (**A**) Representative images (left) and quantifiation (right) of ESCC cell colonies, as determined by colony formation assay after transfection. The over-expression of miR-495 significantly reduced the colony formation number of ESCC cell lines. Conversly, down-regulation of miR-495 improved the colony formation number of ESCC cell lines (**B**) Representative images (left) and quantifiation (right) of flow cytometry analysis of ESCC cells after transfection. Cell cycle analysis revealed that miR-495 has influence the proliferation of ESCC cells by regulating its cell cycle. (**C**) Representative images (left) and quantifiation (right) of migratory and invasive cells per field after transfection. miR-495 significantly depressed the migratory or invasive cells per field.

According to the results of colony formation assays, ectopic miR-495 expression could suppress the proliferation in both ECA109 and TE-1 cell lines. Conversely, down-regulation of miR-495 could promote the proliferation of both cell lines. Consistent with these results, cell cycle analysis revealed that miR-495 arrested cells at the G1/S checkpoint, and ectopic miR-495 expression resulted in an increased G1/G0 population (Figure 2A, 2B).

Similarly, the subcutaneous tumors induced by miR-495 overexpression cells was smaller and grow slower than those induced by scramble cells (Figure 6C). These findings suggests that ectopic miR-495 expression could suppress ESCC cell proliferation *in vivo.*

Then the effects of miR-495 on cell metastasis were explored by performing transwell assays. Transwell assays revealed that miR-495 overexpression reduced the number of cells that moved through the chambers, indicating that miR-495 suppressed migration and invasive ability (Figure 2C).

### MiR-495 binds to the Akt1 3′-UTR and inhibits its expression

**Figure 3:**
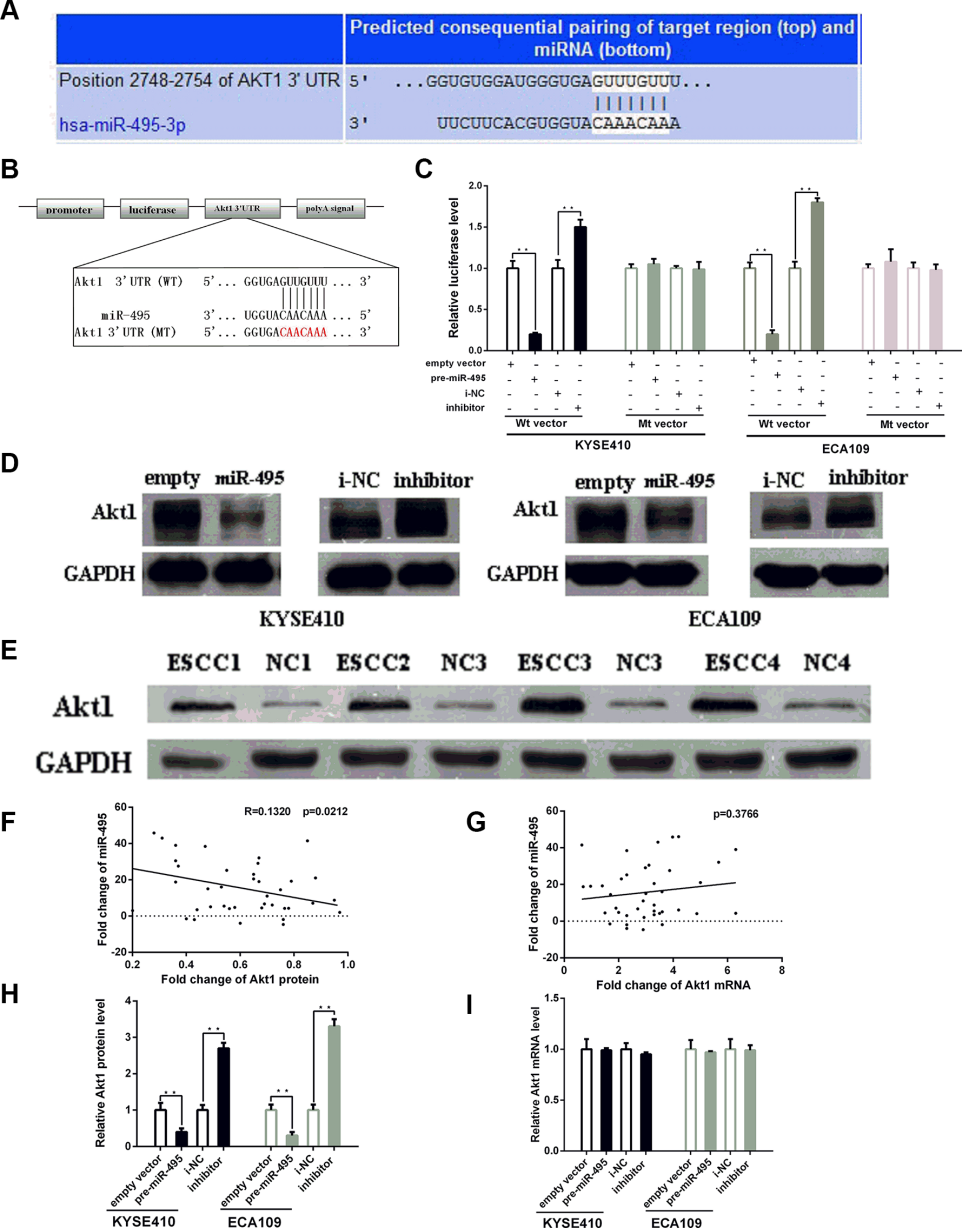
MiR-495 inhibits Akt1 expression by binding its 3′-UTR (**A**) Representations of the predicted target site for miR-495 in the Akt1 mRNA 3′ -UTR. (**B**) Sequence of the miR-495 binding sites within the human Akt1 3′-UTR and a schematic diagram of the reporter constructs showing the Akt1 3′-UTR sequence (WT) and the mutated sequence (MT). (**C**) Direct binding of the miR-495 to Akt1 3′-UTR. KYSE410 and ECA109 cells were transfected using a firefly luciferase reporter containing either wild-type (WT) or mutant (Mut) miR-495 binding sites in the Akt1 3′-UTR with miR-495 overexpression or knockdown. 24 after transfection, the cells were assessed using a luciferase assay kit. The results are displayed as the ratio of firefly luciferase activity in the miR-495-transfected cells to that in the control cells. (**D**) Representative Western blot of Akt1 protein levels in KYSE410 and ECA109 cells after transfection. (**E**) Representative Western blot of paired tumor (ESCC) and normal adjacent tissues(NC). (**F**) Pearson's correlation scatter plot of the fold change of miR-495 and Akt1 protein. (**G**) Pearson's correlation scatter plot of the fold change of miR-495 and Akt1 mRNA. (**H**) Quantitative analysis of the Akt1 protein and levels in KYSE410 and ECA109 cells after transfection. (**I**) Quantitative analysis of the Akt1 mRNA and levels in KYSE410 and ECA109 cells after transfection.

The bioinformatic analysis for target gene prediction identified Akt1 as one of the potential targets of miR-495. The predicted Akt1 3′-UTR binding site for miR-495 is illustrated in Figure 3A.

To further explore whether this site is responsible for miR-495's inhibitory effects, dual luciferase assays were performed. The results showed that the normalized luciferase activity of the WT vector, but not the MT vector, was reduced in the presence of miR-495 (*p* < 0.05) (Figure 3B, 3C). Thus, miR-495 could directly target the seed region of the Akt1 3′-UTR.

Transfection with the miR-495 mimics similarly decreased Akt1 protein levels in both cell lines (Figure 3D, 3H). In contrast, Akt1 protein levels were higher in ECA109 and KYSE-410 cells treated with miR-495 inhibitor than in cells treated with NC. We next examined the Akt1 expression levels in human tumor tissues and paired normal tissues. Western blots demonstrated that all the expression levels of Akt1 in tumor tissues were higher than that in normal tissues (Figure 3E). Bivariate correlation analysis suggested a negative correlation between miR-495 expression and Akt1 protein levels (Figure 3F).

The studies about the expression levels of Akt1 mRNA in ESCC cell lines showed that, Akt1 mRNA expression was not obviously affected by the up- or down- regulation of miR-495 (Figure 3G and 3I). These studies indicated that miR-495 may suppress Akt1 protein levels by inhibiting translation rather than by affecting Akt1 mRNA stability.

### Akt1 is the key mediator of the influence of miR-495 on ESCC cells viability

**Figure 4:**
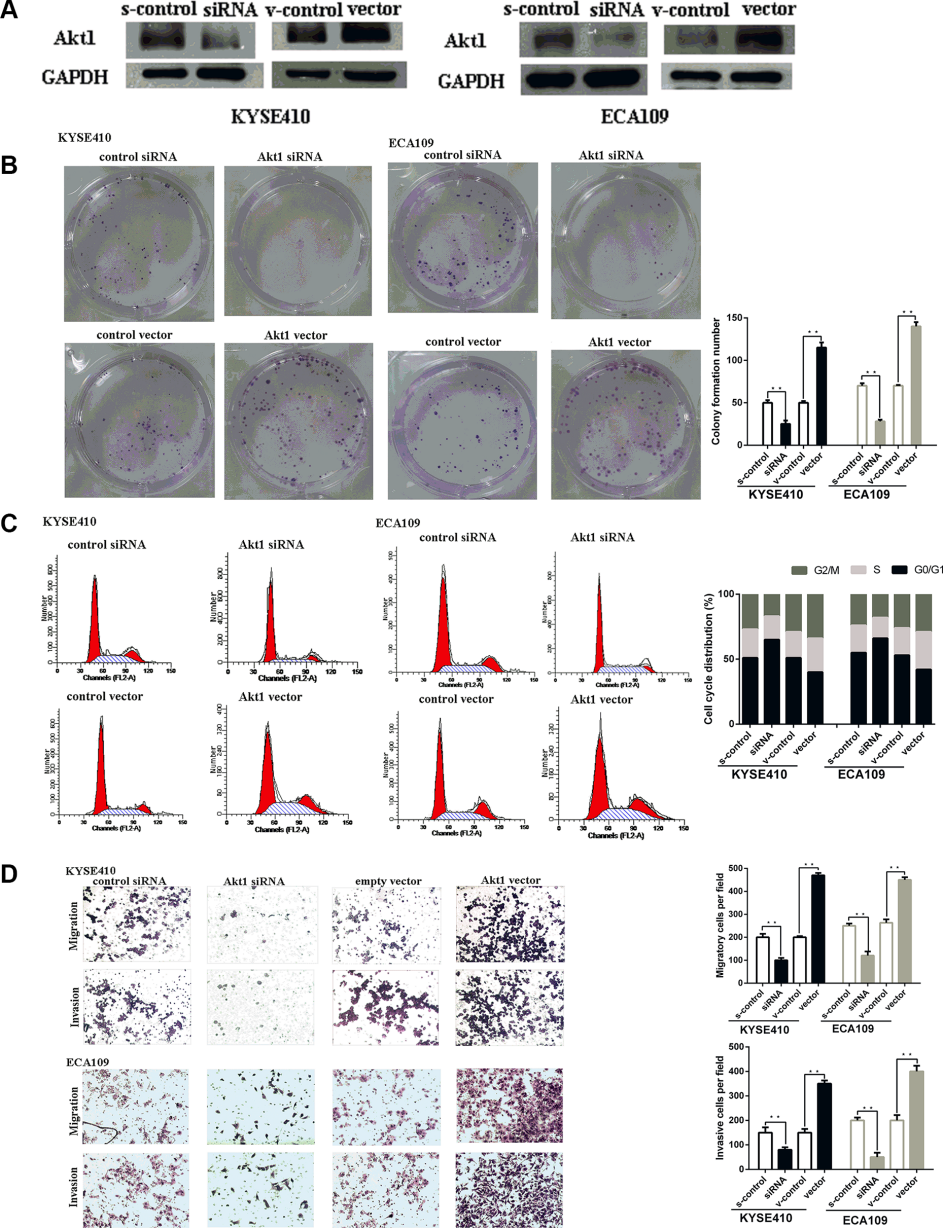
Akt1 promote ESCC progression *in vitro* (**A**) Representative images of western blot analysis of the Akt1 protein level in ESCC cells after transfection. The expression level of Akt1 protein was significantly improved in cells transfected with Akt1 vector and depressed in cells transfected with siRNA (**B**) Representative images (left) and quantifiation (right) of ESCC cell colonies, as determined by colony formation assay after transfection. The over-expression of Akt1 protein significantly improved the colony formation number of ESCC cell lines. Conversly, down-regulation of Akt1 protein significantly reduced the colony formation number of ESCC cell lines (**C**) Flow cytometry analysis of ESCC cells after transfection. Cell cycle analysis revealed that Akt1 has influence the proliferation of ESCC cells by regulating its cell cycle. (**D**) Representative images (left) and quantifiation (right) of migratory and invasive cells per field after transfection. miR-495 significantly depressed the migratory or invasive cells per field.

To explore whether miR-495 inhibited ESCC cell viability by directly targeting STAT3, we adopted a “rescue” methodology. First, 48 hours after the plasmid or siRNA were transfected into ESCC cells, Akt1 protein levels were measured by western blot (Figure 4A). As expected, Akt1 expression promoted ESCC cells growth and metastasis. In contrast, inhibition of AKT1 had the opposite effects (Figure 4B, 4C, 4D).

**Figure 5:**
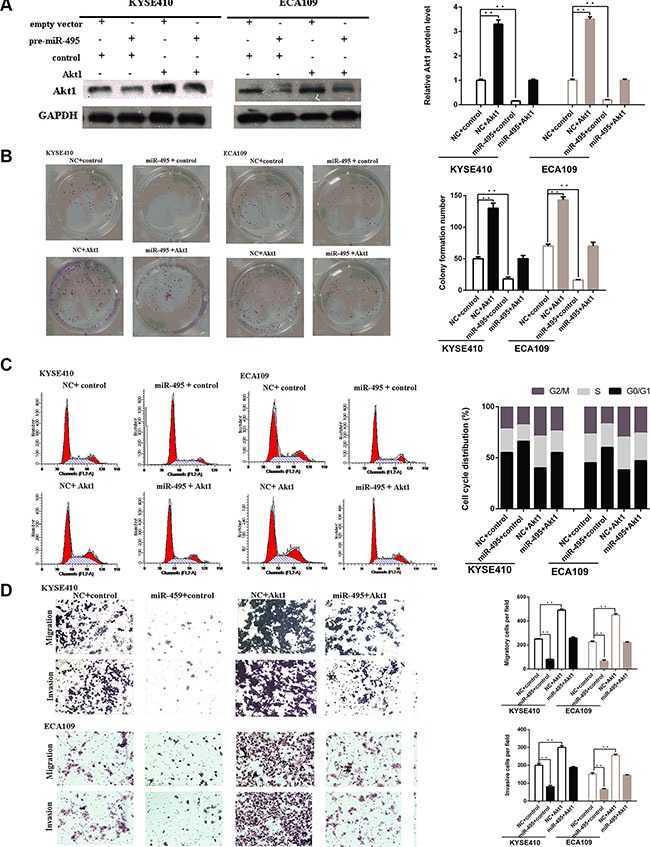
Akt1 are involved in the role of miR-495 in regulation of ESCC cell activity (**A**) Representative images of western blot analysis of the Akt1 protein level in ESCC cells after transfection. The expression level of Akt1 protein in cells transfected with plasmid were higher than that of the negative control, even with suppression by miR-495. (**B**) Representative images (left) and quantifiation (right) of ESCC cell colonies, as determined by colony formation assay after transfection. The suppressive effects of miR-495 on cell proliferation has been partially attenuated by the expression of Akt1. (**C**) Flow cytometry analysis of ESCC cells after transfection. The effects of miR-495 on cell cycle has been partially abolished by the expression of Akt1. (**D**) Representative images (left) and quantifiation (right) of migratory and invasive cells per field after transfection. The effects of miR-495 on cell migration and invasion has been partially abolished by the expression of Akt1.

Finally, we examined whether miR-495 inhibits ESCC cell proliferation, migration and invasion by suppressing Akt1 expression. Western blots demonstrated that the Akt1 protein expression were significantly upregulated in cells treated with plasmid compared with those treated with the negative control, even though miR-495 was suppressed in these cells (Figure 5A). Moreover, restoration of Akt1 levels partially abolished the suppressive influence of miR-495s on cell growth and metastasis (Figure 5B, 5C, 5D). Hence, our results suggest that the tumor-suppressing effects of miR-495 in ESCC might be a consequence of its ability to decrease Akt1 expression.

**Figure 6:**
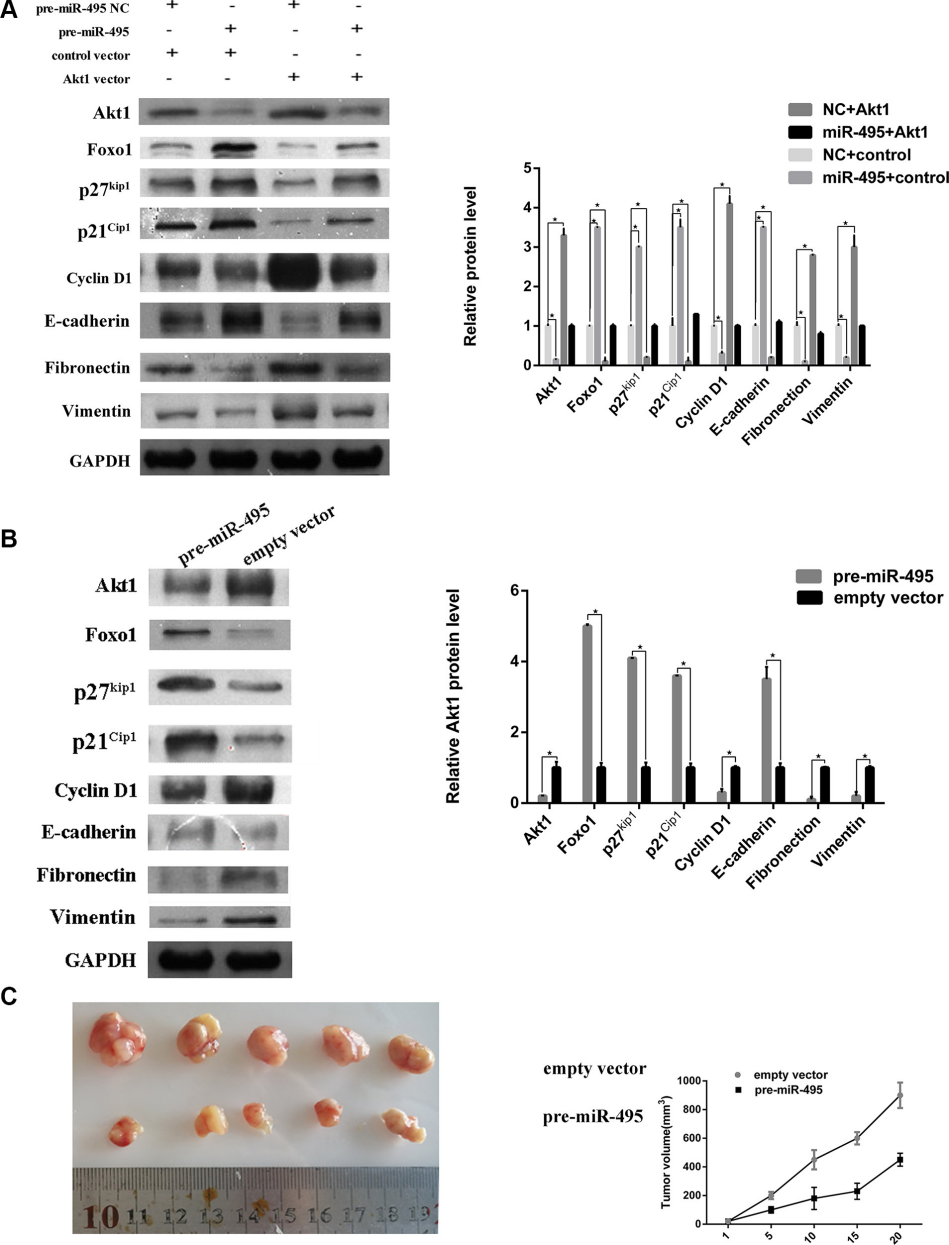
miR-495 represses cell cycle signaling, inhibits epithelial-mesenchymal transition by directy targeting Akt1 to suppress ESCC progression *in vitro* and *vivo* (**A**) Representative images (left) and quantifiation (right) of western blot analysis of relative proteins in ECA109 cell lines. (**B**) Representative images (left) and quantifiation (right) of western blot analysis of relative proteins in the tumors from nude mice. (**C**) miR-495 inhibits HCC cell growth *in vivo*. The HCC mouse model in mice was constructed by using ECA109 cells infected with control vector or miR-495 lentivirus. The size of subcutaneous tumors and local liver tumors in these two groups was calculated and compared.

### miR-495 regulate the cell cycle distribution and epithelial-mesenchymal transition by directly targeting Akt1

As shown above, Akt1 levels are associated with cell cycle distribution. To further explore whether miR-495 induced G0/G1 arrest by regulating the cell cycle signaling pathway, we measured levels of other proteins involved in this pathway. The proteins Foxo1, p27^kip1^, p21^Cip1^, and Cyclin D1 were also regulated by miR-495 and Akt1 (Figure 6A).

Epithelial-mesenchymal transition (EMT) is the major mechanism of tumor cell invasion and migration. We examined the levels of epithelial and mesenchymal markers in ESCC cell lines transfected with miR-495 or Akt1. Western blots revealed that the mesenchymal markers (Fibronectin and Vimentin) and epithelial markers (E-cadherin) were also regulated by miR-495 and Akt1. The western blots results of tumors from mice also demonstrated the influence of miR-495 on cell cycle and EMT signaling pathway (Figure 6B). Our findings suggested that miR-495 and Akt1 may regulate ESCC cell invasion and migration through regulating the EMT signaling pathway.

## DISCUSSION

Altered miRNA expression has been reported to be closely related with the initiation and progression of cancer by activating or inhibiting various processes [15, 16]. More recently, several specialized miRNAs have been demonstrated to be closely related with tumor growth and metastasis [17]. Characterization of miRNAs involved in the progression of ESCC and their targets may contribute to the identification of new prognostic markers and therapeutic targets. Here, we demonstrated that miR-495 was down-regulated in ESCC tissues in comparison with paired non-tumor tissues. Additionally, low miR-495 expression was associated with increased lymph node metastasis (LNM), increased invasion, later TNM stages, and poor overall survival.

We also comprehensively explored the influence of miR-495 on malignancy in ESCC cell lines. As expected, miR-495 overexpression significantly suppressed cell proliferation by inducing the cell cycle arrest in cell lines and tumors resulting from their injection into mice. Moreover, miR-495 transfection also dramatically decreased ESCC cell metastasis *in vitro.* Our findings demonstrate that miR-495 can inhibit the progression of esophageal cancer.

A recent study demonstrated that miR-495 decreases cell proliferation and tumor angiogenesis by inhibiting the expression and activity of Runt-related transcription factor 3 (RUNX3) in gastric cancer cells [11]. To further explore the mechanisms underlying miR-495-mediated inhibition of ESCC cells ability, we examined whether miR-495 targeted Akt1. Akt1 is the crucial mediator of the PI3K/Akt signaling pathway and mediates various cellular functions in a number of tumor types, including gastric cancer [18], glioma [19], lung cancer [20], and ESCC [21]. In glioma patients, overexpression of Akt protein is correlated with later glioma grade and poor prognosis [22, 23]. In ESCC patients, increased AKT1 activity was an independent predictor of survival, with higher p-AKT1 expression linked to poorer OS [21].

Previous studies have demonstrated the major role of miRNA in the regulation of the PI3K/Akt pathway in tumor initiation and progression [18]. Here, we demonstrated that miR-495 could inhibit ESCC cell growth and metastasis *in vitro* and *in vivo* by downregulating Akt1. Akt1 overexpression partially reversed the miR-495-mediated inhibition of ESCC cells ability. Our results demonstrated the anti-tumor effect of miR-495 and that miR-495 might be a useful prognostic marker for ESCC.

Decreases in Akt1 levels mediate EMT and effect cell cycle by regulating proteins such as Foxo1, MMP2, p27^Kip1^ and p21^Cip1^ in cervical carcinoma and glioma [24, 25]. Our results suggest that miR-495 could regulate the protein levels of Foxo1, p27^kip1^, p21^Cip1^, and Cyclin D1, all of these genes are downstream of Akt1, and subsequently inhibit cancer cell growth by promoting cell cycle arrest. Foxo1 is one of the forkhead transcription factors with a highly conserved DNA-binding domain. Overexpression of Foxo1 proteins can activate the cell cycle inhibitors, such as p27^kip1^ and p21^Cip1^, and suppress cell cycle promoters, such as cyclin D1, ultimately resulting in G1/S cell arrest [26]. Similarly, we found that miR-495 decreased Cyclin D1 and increased Foxo1, p27^kip1^, and p21^Cip1^ levels by reducing Akt1 protein levels. A previous study demonstrated that Akt overexpression induces EMT by upregulating the mesenchymal cell-specific markers, including Vimentin and Fibronectin, and downregulating epithelial cell-specific markers, including E-cadherin, in squamous cell carcinoma lines [27]. We therefore examined the role of miR-495 on EMT, a central mediator in tumor metastasis. Restoring Akt1 levels in cells overexpressing miR-495 attenuated the latter's effects on mesenchymal cell-specific markers (Vimentin and Fibronectin) and epithelial cell-specific markers (E-cadherin).

In the present study, miR-495 has been demonstrated to be downregulated in ESCC tumor specimens and was negatively correlated with Akt1 protein levels. In addition, ectopic miR-495 expression inhibited ESCC cell proliferation, migration, and invasion through direct targeting of Akt1 and subsequently altering downstream protein levels. Our data suggest that the miR-495 plays a central role in ESCC development and may serve as a novel target for the treatment of ESCC.

## MATERIALS AND METHODS

### Patients and tissue specimens

Matched surgical ESCC specimens and adjacent non-tumor tissues were obtained from 40 patients at Shandong Cancer Hospital. Written informed consent were provided by all patients. All specimens were subjected to pathological examinations for diagnostic confirmation. This study was performed under the approval of the Ethics Committee of the Shandong Cancer Hospital.

The paraffin-embedded (FFPE) tumor tissues of 43 ESCC patients were collected for miRNA analysis. All patients have received a close follow-up observations for disease recurrence at more than 3-month intervals during the first 2 years after the treatment and more than 6 months thereafter.

### miR-495 target gene prediction

miRNA target prediction was performed with four publicly available algorithms: TargetScan [28], miRanda [29], and PITA [30].

### Cell culture

All the cell lines including human normal esophageal cell line (HEEC) and three human ESCC cell lines (ECA109, KYSE410, and TE-1) were purchased from the Shanghai Institute of Cell Biology, Chinese Academy of Sciences. Cells were cultured in the growth media (DMEM supplemented with 10% FBS) (Gibco, USA) at 37°C under a humidified atmosphere containing 5% CO_2_.

### Quantitative RT-PCR

The miRNeasy Mini Kit (QIAGEN, Germany) was used to isolate the total-RNAs from fresh tissue and miRNeasy FFPE Kit (QIAGEN, Germany) was used to isolate total-RNAs from FFPE tissue samples. Reverse transcription was performed using a miScript II RT Kit (Qiagen, Denmark). qRT-PCR was performed using the miScript SYBR Green PCR Kit and miScript Primer PCR Assay (Qiagen). U6 (RUN6B) was used for normalization. The 2-ΔΔCt method was used to evaluate the relative miRNA expression levels for cells and the 2-ΔCt method was used to evaluate the relative miRNA expression levels for for tissue samples [31].

The SYBR Green PCR Mix (Aidlab, Beijing, China) was used to quantify the expression levels of Akt1 mRNA. GAPDH was used as the internal control and the expression of mRNA were quantified using the 2-ΔΔCt method [31]. The sequences of these primers are listed in Table 3.

**Table 3:** Primers used in this study

Primers	Sequences (From 5′ to 3′)
**GAPDH-F**	TCAACGACCACTTTGTCAAGCTCAGCT
**GAPDH-R**	GGTGGTCCAGGGGTCTTACT
**U6-F**	CTCGCTTCGGCAGCACATATACT
**U6-R**	RACGCTTCACGAATTTGCGTGTC
**U6-RT**	AAAATATGGAACGCTTCACGAATTTG
**MiR-495-F**	GCGAAACAAACATGGTGC
**MiR-495-R**	GCAGGGTCCGAGGTATTC
**MiR-495-RT**	GTCGTATCCAGTGCAGGGTCCGAGGT ATTCGCACTGGATACGACAAGAAGT
**Akt1-F**	CTGAGATTGTGTCAGCCCTGGA
**Akt1-R**	CACAGCCCGAAGTCTGTGATCTTA

### Plasmid construction and oligonucleotide transfection

The expression plasmid (pReceiver-M98-Akt1) of Akt1 protein, the small interfering RNAs (siRNA) targeting human Akt1, miR-495 mimics, miR-495 inhibitor and negative control duplex (NC) were all generated by FulenGen (Guangzhou, China). Cell transfection were performed using lipofectamine 2000 Reagents (Invitrogen, USA). The transfection efficiency of plasmid and siRNA were assessed by RT-PCR and inverted fluorescent microscopy. The transfection efficiency of mimics and inhibitor were assessed by qRT-PCR or western blot, respectively.

### Lentivirus constructs and animal experiments

Lentiviral construct containing pre-miR-495 was packaged using the ViraPower Mix (Invitrogen, Carlsbad, CA). The pre-miR-495-expressing lentivirus was used to stably transfect ESCC cells (ECA109) to construct the miR-495-overexpressing cell line. Empty vector-transfected cells were used as controls.

In order to construct the ESCC xenograft model, 2 × 10^6^ ECA109 cells stably overexpressing miR-495 were inoculated subcutaneously in the BALB/c nude mice's hind limb (Bioscience, China). After 25 days, tumor tissues in mice were resected. Tumor volume was calculated with the formula: length × width^2^ × 1/2.

### Dual luciferase assay

To construct a pGL3-Akt1-3′-UTR-Wt vector (Wt vector), the 3′-untranslated regions (3′-UTR) of Akt1 mRNA was amplified and inserted into the luciferase reporter plasmid. For pGL3-Akt1-3′-UTR-Mut vector (Mt vector), the predicted binding region of miR-495 were mutated (Figure 2D). The reporter construction (Wt vector, Mt vector) were co-transfected with corresponding miR-495 mimics, inhibitor, or NCs and the internal control vector (pRL-tk) using Lipofectamine 2000 (Invitrogen). 48 hours later, the Dual-Luciferase Reporter Assay System (Promega, USA) was used to analyze the luciferase activity which was normalized to Renilla luciferase.

### Western blot

48 hours after various treatments (Thermo, USA), cells were scraped from the wells. SDS-PAGE were used to separate the proteins. Then the proteins were transferred to a PVDF membranes which were blocked with 5% non-fat milk and incubated with the primary antibodies (Abcam, UK). After washed in TBS for three times, signals were probed with secondary antibody (Icllab, USA). The bands were then visualized by chemiluminescence (Millipore, MA, USA) after washing in TBS.

### Colony-formation assay

Cells were seeded onto 6-well plates (500 cells/well) and cultured at 37°C in a humidified 5% CO_2_ atmosphere for 2 weeks. The number of colonies were then counted after staining with crystal violet for 20 min.

### Cell cycle analysis

Two days after transfection, cells were fixed with ice-cold 95% ethanol for 24 h and then dyed with propidium iodide/RNase buffer (BD Biosciences, USA). After 30 min of incubation at 4°C while protected from light, the cells were measured by flow cytometry.

### Invasion and migration assay

Invasion and migration assays were carried out in transwell chamber (Millipore, USA) with or without Matrigel (BD Biosciences, USA). 48 hours after transfection, 0.1 mL FBS-free DMEM containing 2 × 10^4^ cells was placed into the upper chamber and 0.6 mL of the DMEM containing 10% FBS was placed into the lower chamber. After 48 hours tumor cells located on the bottom surface of the membrane were photographed and counted after staining with 0.1% crystal violet for 15 min.

### Statistical analyses

All *in vitro* experiments were performed in triplicate and SPSS19.0 for Windows (IBM, USA) were used to conduct the analysis. Wilcoxon pairs tests were used to assess the expression levels of miR-495 in paired tumor tissues and adjacent non-tumor tissues. Pearson correlation coefficients were used to analyze the relationship between Akt1 and miR-495. Kaplan-Meier methods were performed to calculate the survival curves and the log-rank test were used to estimate the difference. One-way ANOVAs were used to conduct the multiple comparisons. Student's *t*-tests were used to measure the differences between independent two-groups. *p* < 0.05 (*) and *p* < 0.01 (**) were considered statistically significant. All data were expressed as means ± standard deviation (SD).

## SUPPLEMENTARY AND FIGURES


