# Supplementary material for: MiR-495 inhibits esophageal squamous cell carcinoma progression by targeting Akt1
Source: Oncotarget. 2016 Jun 13;7(32):51223–36. doi: 10.18632/oncotarget.9981 (PMC5239471; doi:10.18632/oncotarget.9981)
Supplement: Supplementary file 1 [file oncotarget-07-51223-s001.pdf]

MiR-495 inhibits esophageal squamous cell carcinoma progression by targeting Akt1

Supplementary Materials

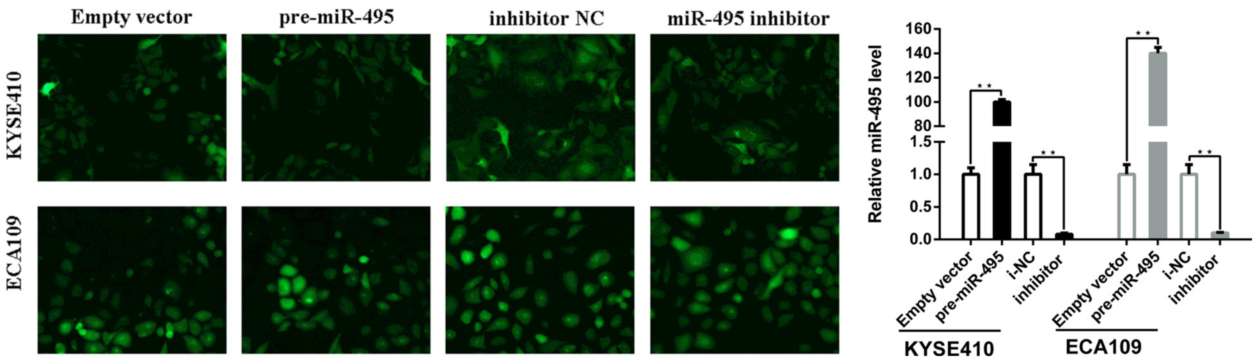

Supplementary Figure S1: Transfection efficiency of miR-495 mimics or inhibitors. Representative images (left) and quantifiation (right) of miR-495 transfection efficiency. Intracellular miR-495 levels increased dramatically in cell lines transfected with mimics and decreased dramatically in cell lines transfected with inhibitors.
